# Stunting and its association with education and cognitive outcomes in adulthood: A longitudinal study in Indonesia

**DOI:** 10.1371/journal.pone.0295380

**Published:** 2024-05-06

**Authors:** Esta Lestari, Adiatma Siregar, Achmad K. Hidayat, Arief A. Yusuf

**Affiliations:** 1 Doctorate in Economics Program, Department of Economics, Faculty of Economics and Business, Universitas Padjadjaran, Bandung, West Java, Indonesia; 2 Research Center for Behavioral and Circular Economics, National Research and Innovation Agency (BRIN), Jakarta, Indonesia; 3 Center for Economics and Development Studies, Department of Economics, Faculty of Economics and Business, Universitas Padjadjaran, Bandung, West Java, Indonesia; 4 SDGs Center, Universitas Padjajaran, Bandung, West Java, Indonesia; University of Waterloo, CANADA

## Abstract

**Background:**

Stunting is associated with adverse outcomes in adulthood. This article specifically aims to analyse the relationship between childhood stunting and education as well as cognitive outcomes for adults in Indonesia.

**Methods:**

Pooled data from wave one (1) and two (2) of the Indonesia Family Life Survey (IFLS) in 1993 and 1997 identified a sub-sample of 4,379 children aged 0–5 by their height-for-age (HAZ) to be compared for their differences in educational outcomes and cognitive abilities in 2014. HAZ was used to proxy relative height to determine stunting status based on 2006 WHO child’s growth standards. Education and cognitive abilities outcomes include years of schooling, age of school entry, grade repetition, and scores for cognitive and math tests. The study employs estimation models of pooled regressions and instrumental variable (IV) to address problems of endogeneity and bias from omitted variables.

**Results:**

Stunting and relatively small stature had significant associations with cognitive development, and they worked as intermediaries to cognitive developmental barriers as manifested in reduced educational outcomes. A lack of one SD in HAZ was associated with 0.6 years shortened length of the school, 3% higher chances of dropouts from secondary school, and 0.10–0.23 SD lowered cognitive and numerical scores. Similarly, stunting is associated with decrease cognitive test scores by 0.56–0.8 SD compared to non-stunting, two years less schooling, and 0.4 years of delayed entry to school. As for cognitive abilities, stunting is associated with lower cognitive and numerical abilities by 0.38–0.82 *z*-scores.

**Conclusion:**

Growth retardation during childhood in Indonesia was associated with lower cognitive abilities, particularly during school age, and this correlation faded as individuals grew up. Subsequently, growth retardation is significantly linked to lower educational outcomes. Impaired growth has implications for reduced lifetime earnings potential mediated by diminished cognitive capacity and lower educational attainment. The finding suggests that development in Indonesia during recent decades has not provided an adequate environment to enable children to achieve their potential educational outcomes.

## Introduction

Stunting is one of six forms of malnutrition prioritised to be eradicated by 2025 [1]. That happened to more than 149.2 million children under five in 2020, whereas Asia and Africa shared the largest burden at 54% and 40%, respectively [2]. Despite being a middle-income country and a member of the G20, Indonesia continues to struggle with malnutrition, with 30.8% of children under five experiencing stunted growth in 2018 [3]. According to the World Health Organization (WHO), Indonesia was among the countries with the highest rates of stunting in the world, with a rate slightly better than Cambodia (32.4% in 2014) and Lao PR (33.1% in 2017) in the region [2].

As a marker of long-term chronic malnutrition, stunting has numerous adverse consequences, including impaired physical and cognitive development, low educational achievement [4–9], a reduction in lifetime income [10], an increased risk of non-communicable diseases and poor birth outcomes for the future generation [11]; leads to decrease chances of escaping poverty [12]. Therefore, addressing stunting is crucial for improving health outcomes and building a supportive socio-economic environment that allows children to reach their full potential [13, 14].

Specifically, empirical studies showed that growth retardation or stunting has been associated with adverse educational outcomes such as shorter years of schooling, lower cognitive abilities, delayed school entry, and a higher risk of failing grades in countries such as Brazil, Guatemala, India, South Africa, the Philippines, Vietnam and Ethiopia [15–18]. Grantham-McGregor et al. (2007) found that stunting at 24 months of age was linked to a 0.9 years delay in school entry and a 16% increased risk of failing grades [19]. Another study by Hoddinott et al. (2013) reported that an increase in height-for-age z-score (HAZ) was related to more extended schooling, higher test scores for reading, and nonverbal cognitive abilities [20].

From the mid-1990s until the end of the New Order regime in 1990, Indonesia was one of the most rapidly growing Asian countries. However, in the following two decades, the high economic growth was not accompanied by sufficient human capital investment. The World Bank (2020) estimated that Indonesia’s productive labour would only reach 53% of its potential, partly due to poor childhood nutritional status [21]. This indicates that the high economic growth experienced by Indonesia until recently may not necessarily be accompanied by improved human capital quality.

To the best of our knowledge, studies on the impact of stunting in Indonesia are still relatively limited, particularly with regard to educational outcomes, despite a relatively high number of studies on determinants and interventions of stunting [22–29]. The challenges come from the requirement for reliable longitudinal data capable of capturing the developmental trajectory of children into adulthood. Another challenge in studies in this area is the estimation techniques that can minimise measurement errors and endogeneity commonly found in health and education studies. Referring to Behrman (1997) [30], one of the most common failures is the exclusion of the possibility that health might be endogenous, which leads to measurement problems and potentially biased estimates of the relationship between the child’s health and education. The bias may arise primarily from unobserved household or community characteristics omitted in the estimations.

This study aims to fill the gap in Indonesian literature on the consequences of growth failure and provide a robust estimation of the linkage between chronic childhood malnutrition (stunting) and adults’ educational outcomes. By applying instrumental variables, this study demonstrates a reliable estimate that controls bias from heterogeneity in households and communities and endogeneity problems between childhood health measurement and academic outcomes. The goal is to identify potential interventions that may improve educational outcomes for stunted individuals and quantify the strength of the relationship between stunting and academic outcomes.

## Methods

This study utilises The Indonesian Family Life Survey (IFLS) data, a longitudinal data spanned over 21 years from 1993 to 2014 covering a representative sample of 83% of the population and over 30,000 individuals across 13 provinces in Indonesia. The survey collected data on individual respondents, their families, their households, and the communities in which they live on various aspects of social, economic, and health issues, including educational achievements and cognitive abilities Specifically, this study employs data from waves 1–2 (1993 and 1997) [31, 32] as the baseline and waves 3–5 (covering the years beyond 1997 up to 2014) for the outcome variables [33–35]. Data from the IFLS surveys are publicly available for those who have registered their interest on the RAND Corporation website [36].

### Study population

The sampling frame for this study consists of combined data for children aged 0–5 years from IFLS wave 1 (1993) and wave 2 (1997) who had complete records of height, weight and age. The follow-up data encompassed multiple waves and allowed us to examine the outcome of interest in 2000, 2007, and 2014 (wave 3–5). Pooled data identified sub-sample of 4,379 children below five years old to be estimated.

### Variables characterisation

#### Characterisation of stunting and relative height

We calculated the child’s height-for-age (HAZ) as a z-score using the age and sex-specific references from the WHO growth standard based on their height for children <5 years [37]. Children with a height-for-age-z-score of <-2 were categorised as stunted [38] and used as the reference for dummy variables of status for stunting. For this purpose, one was assigned as the value when the HAZ was <-2, or stunting, and zero was assigned when the HAZ was >-2, or not stunting. Children above two years old were measured in a standing position, and those below the age were measured in lying down otherwise, there was a 0.7cm adjustment following the WHO measurement standards [39].

**Fig 1:**
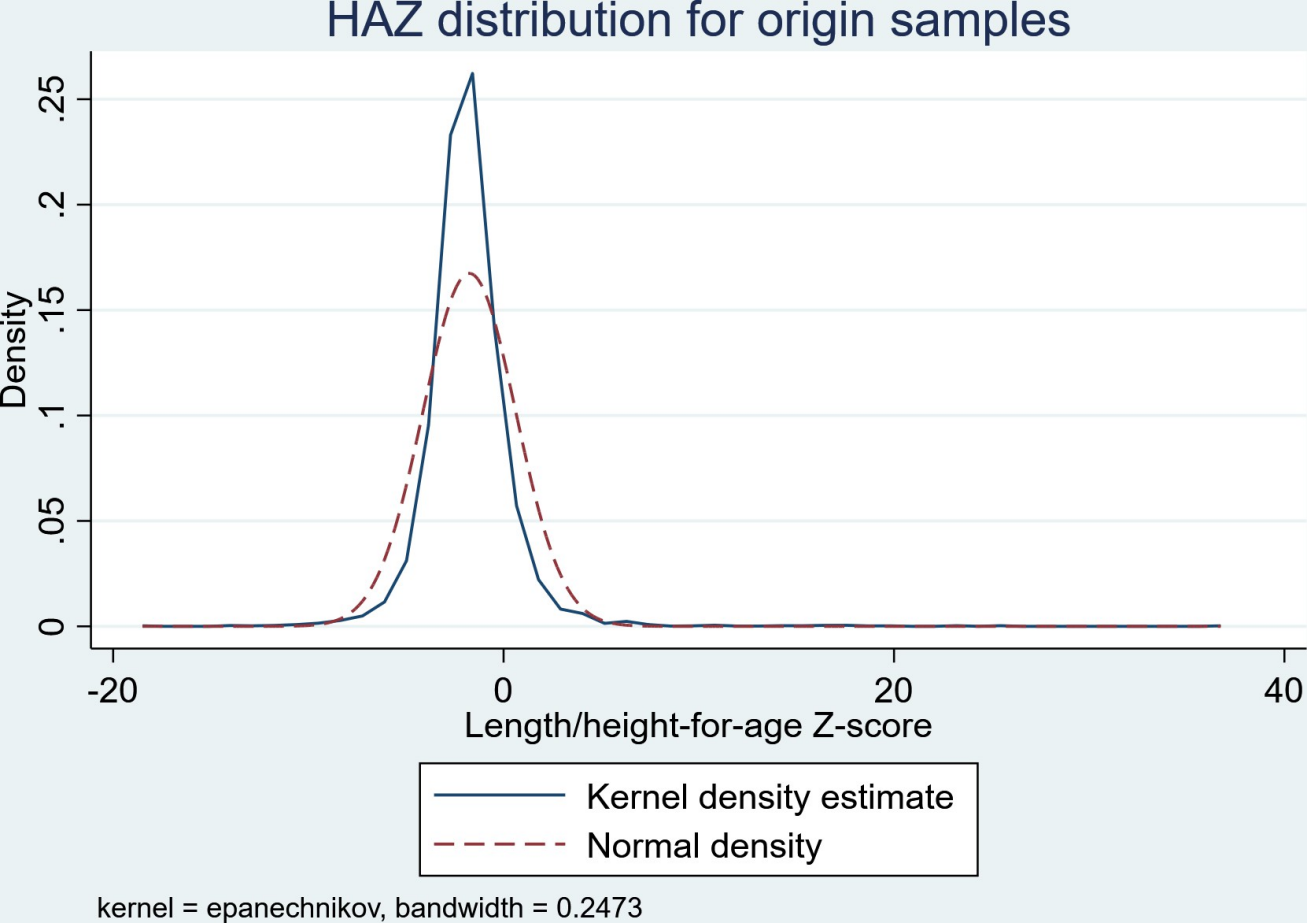
The distribution of HAZ data for the original sample.

The dataset is pooled data of height-for-age collected in 1993 and 1997 for children under the age of five with a total sample of 5,224. Some of the children initially identified in wave 1 were still below the age of 5 in wave 2, two years later. To avoid counting them twice, we excluded them in the sample (n = 349). By this, we have ensured that the count reflects unique children, and they were not double-counted, resulting in 4,875 children. The original dataset distribution can be seen in Fig 1.

**Fig 2:**
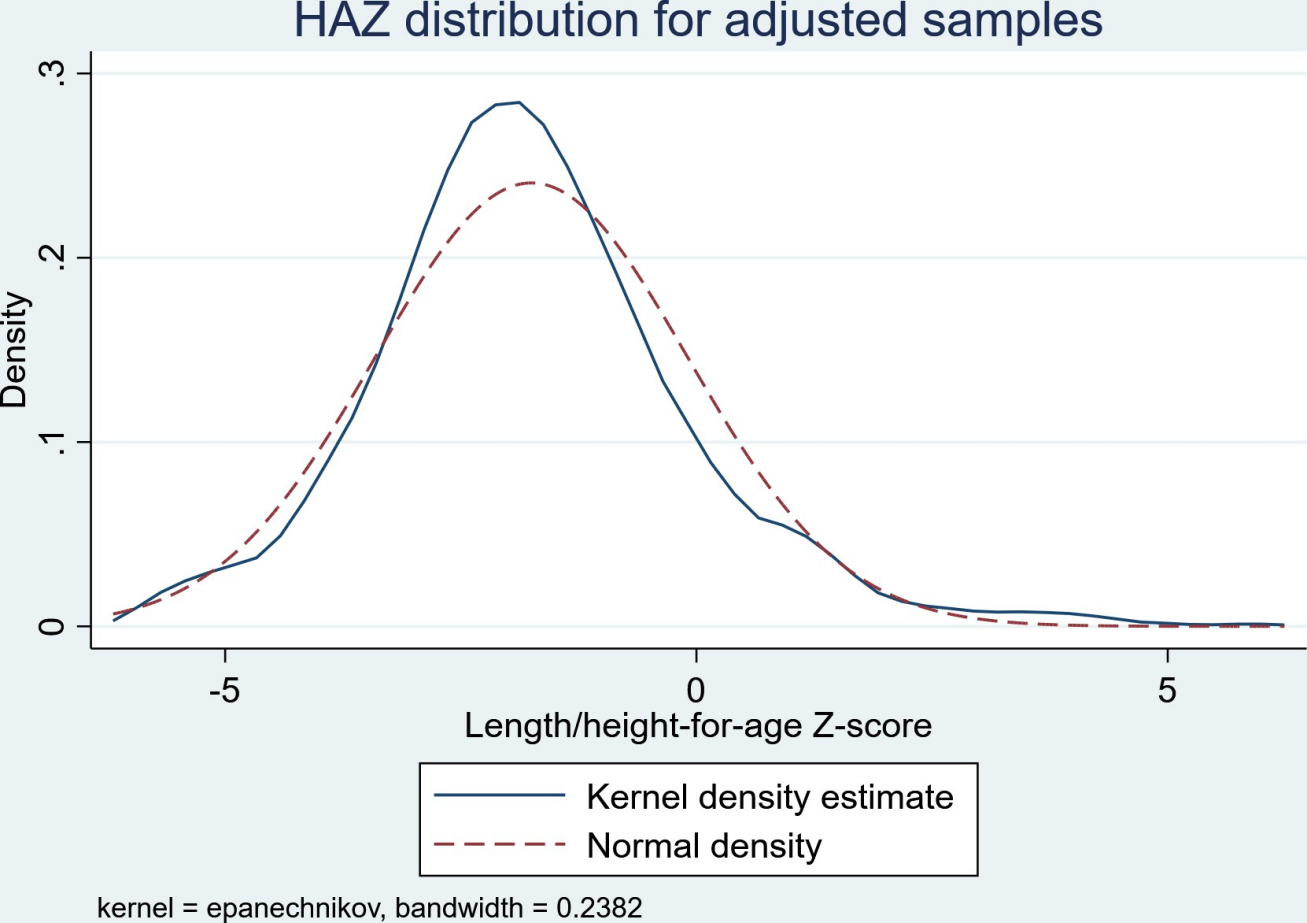
The distribution of HAZ data for the original sample.

However, due to significant measurement errors associated with height mismeasurement, we excluded 496 children from the sample. This outlier specifically comes from the inconsistencies in height measurements relative to the subject’s ages. Following Alderman’s study (2006), we utilized samples with HAZ values falling within the range of -6 to +6 to ensure data quality. The children were then tracked down until they were aged 17–26 years in 2014, and there remained a total of 4,379 respondents. The distribution of adjusted sample is outlined in Fig 2.

The follow-up surveys in Waves 3 (2000), 4 (2007), and 5 (2014) aimed to assess the educational and cognitive outcomes of the participating children. Child samples of 4,379 may not always be included in the last three waves, primarily because some outcome variables require specific age criteria or they are no longer traceable in a particular wave. However, excluded children in a wave can be included again into the estimates if they become traceable and their outcome data is found in subsequent waves.

Using the methods described in Fitzgerald, Gottschalk, and Moffitt (1998) [40] and Alderman et al. (2001) [41], we estimated a probit to determine if there was attrition based on observable characteristics, as presented in Table 1. As part of this, a dependent variable equal to 1 if the school achievement is observed in 2004 and 0 otherwise is regressed along with height-for-age and a variety of child and family characteristics. Because there was no statistically significant link between height-for-age and attrition for all outcomes except for adults’ cognitive abilities thus, there is not enough evidence of attrition bias.

**Table 1:** Testing for selective attrition using Fitzgerald, Gottschalk, and Moffitt method.

Outcomes measured in 2014							
Exposures	Childhood cognitive z-score	Adolesc. cognitive z-score	Adult. cognitive z-score	Age started school (years)	Grade retention	Dropout school	Years of school (years)
	(1)	(2)	(3)	(4)	(5)	(6)	(7)
Initial HAZ	0.02	0.01	-0.04***	-0.01	-0.01	-0.01	-0.00
	(0.02)	(0.02)	(0.01)	(0.01)	(0.01)	(0.04)	(0.01)
Child is boy	-0.14**	-0.05	-0.25***	-0.19***	-0.10**	-0.17	-0.18***
	(0.06)	(0.07)	(0.05)	(0.05)	(0.04)	(0.13)	(0.05)
Household size	-0.04***	-0.00	-0.01***	-0.01	-0.00	0.00	-0.00
	(0.01)	(0.01)	(0.01)	(0.01)	(0.01)	(0.02)	(0.01)
Asset index	0.18***	0.08**	0.11***	0.04	0.12***	0.08	0.04
	(0.041)	(0.04)	(0.03)	(0.03)	(0.03)	(0.09)	(0.03)
Rural/Urban	-0.04	0.02	0.07	0.11**	0.21***	0.15	0.10**
	(0.07)	(0.06)	(0.05)	(0.05)	(0.05)	(0.13)	(0.05)
Child from Java	0.11	0.03	-0.05	0.00	-0.10*	-0.31*	0.00
	(0.08)	(0.07)	(0.06)	(0.06)	(0.06)	(0.18)	(0.06)
Child from Sumatra	-0.08	-0.06	-0.02	-0.07	-0.07	-0.23	-0.08
	(0.09)	(0.08)	(0.07)	(0.07)	(0.06)	(0.21)	(0.07)
Maternal educ. Level	-0.12*	-0.21***	-0.11**	-0.06	0.13***	0.24	-0.08
	(0.07)	(0.06)	(0.05)	(0.05)	(0.05)	(0.18)	(0.05)
Recurrent age	0.62***	-0.10***	-0.25***	-0.05***	-0.17***	-0.01	-0.05***
	(0.02)	(0.01)	(0.01	(0.01)	(0.01)	(0.03)	(0.01)
Constant	-12.88***	3.69***	5.90***	1.93***	3.81***	2.46***	2.02***
	(0.43)	(0.33)	(0.27)	(0.26)	(0.26)	(0.7)	(0.26)

Standard errors in parentheses

*** p<0.01

** p<0.05

* p<0.1

#### Characterisation of educational outcomes

In the 2014 IFLS, educational outcomes are measured by several variables: years of schooling, grade repetition, binary variable for school dropout, and age of first enrolment. The variable "years of schooling" represents the total number of completed years of education, with a minimum value of 0 for individuals who have never attended school or did not complete primary education and a maximum value of 22 years for those who have completed college or university. The school dropout variable is a binary variable set to one if an individual only completed elementary school and did not continue to secondary school and zero otherwise. The grade repetition is also a binary variable, with a value of one if the individual had any experience of grade repetition until 2014. Finally, the age of the first enrolment to school is the age at which a child was first admitted to elementary school.

IFLS provides a cognitive capacity section to measure the level of intellectual development using Raven’s Progressive Colored Matrices (RPM) method and mathematics test.

The Raven test is considered a valid measure of cognitive ability due to its strong theoretical foundation, robust psychometric properties, and demonstrated correlations with other intelligence measures, making it suitable for diverse populations [42]. The RPM has been widely used as a cognitive ability indicator for studies conducted in Indonesia [26, 43, 44], as well as in various other countries, including [45]. RPM measures fluid intelligence from non-verbal cognitive scores and mathematical tests to measure numerical abilities. The levels of tests given to the respondents were divided into an easier version for all respondents aged 7–14 and a more complex version for all respondents aged 15–24 years old. The cognitive and mathematics scores are measured based on the number of correct responses to a set of questions, which are subsequently standardised to obtain the final scores. The scores were retrieved from the 2000, 2007, and 2014 waves to represent the school-age, adolescence, and adulthood phases.

#### Potentially confounding variables

We controlled for individual, parental, and household variables in the base years (1993 and 1997). Individual variables consisted of gender and age in 2014. Parental characteristics are represented by their age in 1993/1997, and a dummy variable is whether the mother was working in the base year. The household characteristics consist of the household size and households’ welfare index in the base year. The welfare index is the assets-based indicator according to the households’ assets as the representative figure of the socio-economic status in the community [46] and is divided into three terciles (poorest, middle, and richest).

Meanwhile, household infrastructure consists of binary electricity, safe drinking water, and sanitation variables. Fixed effects on regional variations are captured by dummy variables of rural/urban and major islands: Java, Sumatra, Borneo (Kalimantan), Sulawesi and Nusa Tenggara. The last three regions are combined into one variable due to their relatively smaller number of respondents than Java and Sumatra. These three categories also represent the population density in Indonesia between the western and eastern parts of Indonesia to represent regional disparity in development.

### Statistical analyses

To determine the association between the potential effect of stunting and relative height at the individual level, multivariate regression (OLS) and instrumental variables estimates were applied. Multivariate least squares were conducted for outcomes with continuous values such as years of schooling, standardised cognitive and math scores, and age-started schooling. Meanwhile, linear probit regressions addressed discrete grade retention and dropout school outcomes. The basic model is as follows:

$$Y_{i}=\beta_{0}+\beta_{1}S_{i}+\sum_{m=14}^{p}\beta_{m}X_{i}+\varepsilon_{i}$$ (1)

In which the dependent variable *Y*_*i*_ consists of the set educational outcomes, *β*_*1*_ is the main effect of nutritional status divided into relative height (height-for-age z-score) and the dummy for stunting, and *X*_*i*_ is the covariate vector consisting of characteristics of children, parents, families and regions.

One problem with using OLS regression is the possible endogeneity of nutritional status (proxied by HAZ and stunting status) with residuals confounded by other unobserved factors correlating with stunting and outcomes [20, 47]. Applying OLS estimation would likely produce omitted variable bias, for example, if unobserved variables (such as parenting skills and parenting time spent) may positively affect early-life nutrition, cognitive skills, and educational outcomes. If there was the case, thus in our model, the unobserved variables for the cognitive abilities and education outcomes would be included in the error term and, with the assumptions specified in the previous sentence, the error term would be positively correlated with nutritional status and other exogenous variables in our model. As a result, the estimated parameters tend to be upward biased [48]. Another approach that leaves the unobserved variable in the error term is using instrumental variable as an estimation method that recognizes the presence of the omitted variable and treats the HAZ and stunting as endogenous. The instrumental variables of nutritional status needs to satisfy two conditions: (1) it should have no partial effect on educational and cognitive outcomes, and it should not be correlated with other factors that affect outcomes. (2) It must be related, either positively or negatively, to the endogenous explanatory variable (relative height and stunting) [49].

#### Selection of instrumental variables

Several instrumental variables (IVs) are commonly used to study the relationship between childhood health and adults outcomes, including the mother’s height [48], the status of being twins [20], environmental variables such as rainfall and vegetation [50], specific randomised interventions [20], or regional variables such as food prices and access to health facilities [51]. In Indonesia, no targeted nutritional interventions were aimed at addressing specific malnutrition conditions prior to 1993. The 1990s were also marked by the New Order regime, during which the production and prices of staple foods were controlled by the central government to achieve food self-sufficiency. Therefore, fluctuations in food prices and nutritional interventions could not be considered potential instrumental variables. Additionally, there is a lack of data on the status of twins in IFLS and no information available on the environment over the past two decades.

In this study, the instrumental variables employed was the birth month dummy variable, divided into August to January, and February to July. The rationale behind using both of these months was grounded in the consideration of two distinct seasons (rainy and dry) and the planting cycle prevalent in Indonesia. August to January is commonly a rainy season and planting period, while in February is the start of dry season. Maccini and Yang (2009) [52] correlate seasonal factors, specifically rainfall, and a range of individual outcomes, such as health, education, and asset indices. Their findings indicated a correlation between rainfall and health, with higher early-life rainfall having substantial positive effects on adult outcomes for women but not for men. These observed patterns are most plausibly attributed to the favorable impact of rainfall on agricultural productivity, resulting in increased household incomes, enhanced food availability, and improved health for infant girls. Building upon this concept, this study adopts the birth month as an instrumental variable for assessing children’s nutritional status.

Specifically, children born between August and January are assigned a value of one (1), while those born between February and July are assigned a value of zero (0). This variable was assumed to be correlated with nutritional status and may be exogeneous with the educational outcomes and therefore meet the conditions as a appropriate instrument. Furthermore, in addition to the birth season, mother heights were also treated as instruments considering the variable brings genetic variation in an individual’s height. Although parental heights were positively correlated with the child’s nutritional status and might also be associated with the child’s cognitive abilities, this relationship is often indirect [48, 53].

### Ethics approval and consent to participate

This study is based on a survey conducted by the Rand Corporation, which has been designed and executed in accordance with the principles of ethical research. The survey has obtained ethical clearance from Institutional Review Boards (IRBs) in the United States and Indonesia at the University of Indonesia (IFLS 1 and 2) and the University of Gadjah Mada (IFLS 3, 4, and 5). The protocol approval number (i.e., ethical clearance number) that RAND’s Human Subjects Protection Committee (RAND’s IRB) gave IFLS5 was s0064-06-01-CR01. All necessary consent requirements for participants, both adults and children, were fulfilled and authorised by the IRBs before the start of data collection. All participants provided their written consent to take part in the survey.

Additionally, consent was obtained from the legal representatives, such as next of kin, caretakers, or guardians, of any children included in the survey on their behalf. This ethical clearance and participant consent process ensures that the data collected is valid and reliable and that the rights and well-being of the participants are protected throughout the survey. Data in this study is de-identified by unique ID numbers, enabling longitudinal tracking while ensuring security and confidentiality.

## Results

**Table 2** reports the summary statistics of all variables of interest. Approximately 47% of children aged 0–60 months were classified as stunted based on their height-for-age (HAZ), with an average HAZ of –3.08 (nearly severe stunting). The average height for children in this age group is 81.3cm, with a difference of over four cm between the stunted and non-stunted groups (85.95cm). The differences in children’s characteristics between the stunting and non-stunting group are statistically significant.

**Table 2:** Descriptive statistics for children’s characteristics and outcomes.

** **	** **	**ALL samples**			**Stunted below five y.o**			**Non-stunted below five y.o**			**Diff.**
** **	** **	**Obs.**	**Mean**	**Conf. interval**	**Obs.**	**Mean**	**Conf. interval**	**Obs**.	**Mean**	**Conf. interval**	
**CHILD LEVEL FACTORS**											** **
Sex	= 1 if male	4,379	0.51	(0.50–0.53)	2,039	0.54	(0.51–0.56)	2,340	0.49	(0.47–0.51)	- 2.71***
Age	Child’s age in 1993 or 1997 (years)	4,379	2.26	(2.21–2.30)	2,039	2.44	(2.38–2.50)	2,340	2.1	(2.04–2.16)	-7.88***
Height	Height in 1993 or 1997 (cm)	4,379	84.08	(83.7–84.5)	2,039	81.93	(81.47–82.39)	2,340	85.95	(85.37–86.53)	10.51***
HAZ^a^	Height-for-age *z* score at age 0–5 years in 1993 or 1997	4,379	-1.75	(-1.80 –-1.70)	2,039	-3.08	(-3.12 –-3.04)	2,340	-0.59	(-0.65 –-0.54)	74.35***
Stunting^a^	= 1 if HAZ at age 0–5 <-2 in 1993 or 1997	4,379	0.47	(0.45–0.48)	2,039	-	-	2,340	-	-	
**PARENTAL FACTORS**											
Maternal education	Mother’s categorical value of the highest education level completed in 1993 or 1997	4,209	0.2	(1.22–1.25)	1,964	0.15	(1.14–1.18)	2,245	0.25	(1.27–1.32)	9.34***
Paternal age	Father’s age in 1993 or 1997 (year)	3,829	34.97	(34.72–35.23)	1,804	35.36	(34.97–35.74)	2,025	34.63	(34.29–34.97)	-2.81***
Maternal age	Mother’s age in 1003 or 1997 (year)	4,212	29.7	(29.51–29.90)	1,967	29.98	(29.69–30.27)	2,245	29.46	(29.20–29.72)	-2.63***
Paternal height	Father’s height in 1993 or 1997 (cm)	3,047	161.26	(161.05–161.47)	1,437	160.18	(159.89–160.47)	1,610	162.22	(161.93–162.51)	9.74***
Maternal height	Mother’s height in 1993 or 1997 (cm)	3,797	150.09	(149.92–150.25)	1,789	148.96	(148.72–149.21)	2,008	151.08	(150.86–151.31)	12.49***
Maternal working	= 1 if the mother worked in the base year	3,916	0.36	(0.34–0.37)	1,847	2.59	(0.35–0.39)	2,069	2.38	(0.32–0.36)	-1.90**
**HOUSEHOLD FACTORS**											** **
Number of household members	#	3,907	7.78	(7.63–7.93)	1,801	7.81	(7.60–8.03)	2,106	7.75	(7.55–7.96)	-0.4
Per capita expenditure	Household per capita expenditure in 1993 or 1997 (000 IDR)	4,371	82.29	(72.9–91.7)	2,037	62.64	(59.6–65.7)	2,334	99.44	(82.1–116.8)	3.83***
Electricity	= 1 if the household utilise electricity in 1993 or 1997	4,376	0.73	(0.72–0.75)	2,038	0.68	(0.66–0.70)	2,338	0.78	(0.76–0.79)	7.10***
Safe drinking water	= 1 if the household had safe source of drinking in 1993 or 1997	4,364	0.93	(0.92–0.94)	2,034	0.91	(0.90–0.92)	2,330	0.95	(0.94–0.96)	5.49***
Sanitation	= 1 if the household has safe defecation 1993 or 1997	4,257	0.34	(0.32–0.35)	1,979	0.27	(0.25–0.29)	2,278	0.39	(0.37–0.41)	8.04***
Wealth index	Household’s wealth index in 1993 or 1997: Poorest = 1; Middle = 2, Richest = 3	4,379	1.67	(1.64–1.69)	2,039	1.63	(1.60–1.66)	2,340	1.7	(1.67–1.73)	2.96***
Rural/Urban	= 1 if urban	4,377	0.4	(0.39–0.42)	2,038	0.33	(0.31–0.35)	2,339	0.47	(0.45–0.49)	8.99***
**EDUCATIONAL OUTCOMES**											
Childhood Raven test score	Raven test score on the 1–100 scale in 2000 (wave 3)	2,323	61.7	(60.70–62.71)	1,086	58.69	(57.22–60.15)	1,237	64.35	(62.98–65.71)	5.55***
Childhood numerical score	Numerical test score on the 1–100 scale in 2000 (wave 3)	2,147	58.02	(56.99–59.04)	997	56.81	(55.33–58.29)	1,150	59.06	(57.63–60.49)	2.14**
Adolescence Raven test score	Raven test score on the 1–100 scale in 2007 (wave 3)	3,893	78.22	(77.60–78.85)	1,808	76.93	(76.00–77.86)	2,085	79.34	(78.50–80.19)	3.77***
Adolescence numerical score	Numerical test score on the 1–100 scale in 2007 (wave 3)	3,787	57.05	(56.18–57.92)	1,762	55.13	(53.86–56.40)	2,025	58.72	(57.52–59.91)	4.03***
Adulthood Raven test score	Raven’s test score on the 1–100 scale in 2014 (wave 5)	3,194	74.73	(73.98–75.47)	1,488	73.04	(71.92–74.16)	1,706	76.19	(75.19–77.19)	4.13***
Adulthood numerical score	Numerical test score on the 1–100 scale in 2014 (wave 5)	3,509	38.26	(37.25–39.26)	1,618	35.53	(34.15–36.90)	1,891	40.59	(39.16–42.02)	4.97***
Failing grades	= 1 if ever failing grades	2,907	0.16	(0.15–0.17)	1,331	0.17	(0.15–0.19)	1,576	0.15	(0.13–0.16)	-1.99**
Age started school	The age first admitted to elementary school (years)	3,304	6.36	(6.33–6.38)	1,534	6.41	(6.37–6.44)	1,770	6.31	(6.28–6.34)	-4.04***
Drop out	= 1 if never proceeded to secondary school	4,329	0.13	(0.12–0.14)	2,017	0.15	(0.13–0.16)	2,312	0.11	(0.10–0.12)	-3.92***
Years of schooling	Years of schooling in 2014	3,362	10.92	(10.82–11.03)	1,553	10.69	(10.54–10.85)	1,809	11.12	(10.98–11.26)	4.03***

95% CIs in parentheses.

^a^Based upon the 2006 WHO Child Growth Standards for children <5 years [37].

*** indicates statistically significant at the 1% level, and

** indicates statistical significance at the 5% level.

Stunted children are more likely to live in rural areas (67%) and in households with less adequate sanitation (73%). Regarding the economic background, families with stunted children tend to have lower per capita expenditure (IDR 62,000) than those without stunting (IDR 99,000). Almost all measured parental characteristics between the stunted and non-stunted groups, such as parental age and height, maternal employment status, and maternal education, also showed statistically significant differences. Household size does not show a statistically significant difference between the two groups.

When reaching adulthood, individuals with a history of stunting are also found to exhibit statistically significant differences in their academic achievements. These individuals tend to have lower cognitive and math test scores during various stages of growth, including school age, adolescence, and adulthood. Additionally, they are inclined to achieve shorter educational attainment, being placed in higher grade levels and having a shorter duration of schooling (10.7 years of education compared to 11.12 years for those without childhood stunting).

### Relative height and educational outcomes

Table 3 reported the estimation results of the relationship between relative height (HAZ) and educational outcomes. The sample size for the estimated outcomes in both OLS and IV regressions varies (S1 Table). The grade repetition and dropout school estimations are performed in linear probit models. Overall, the OLS estimates (Table 3: 1–3) show that relative height (HAZ) is significantly associated with higher educational achievements in various life-course from school to adulthood. Holding other variables constant, one additional z-score would likely increase cognitive (Raven’s) test scores in school-age (0.04-SD) and adolescence (0.03-SD), yet it has no relationship with adults’ cognitive abilities. In contrast, relative height influences individuals’ math ability during adolescence (0.02-SD) and adulthood (0.06), without a relationship during school age.

**Table 3:** Estimation results for relative height and educational outcomes.

	Relative height (HAZ)^1^ when measured prior to age five^2^					
	OLS^a^			Instrumental variables estimates		
Educational outcomes	Coef.	ci	*P*	Coef.	ci	*P*
	(1)	(2)	(3)	(4)	(5)	(6)
Childhood’s Raven (*Z*-scores)	0.04	(0.01–0.08)	0.01	0.22	(0.06–0.40)	0.01
Childhood’s Numerical (*Z*-scores)	0.01	(-0.02–0.04)	0.35	0.11	(-0.07–0.28)	0.09
Adolescence’s Raven (*Z*-scores)	0.03	(0.01–0.05)	0.00	0.10	(0.00–0.20)	0.05
Adolescent’s Numerical (*Z*-scores)	0.02	(0.00–0.04)	0.08	0.12	(0.01–0.24)	0.03
Adult’s Raven (*Z*-scores)	0.02	(-0.01–0.04)	0.25	0.02	(-0.13–0.17)	0.81
Adult’s Numerical (*Z*-scores)	0.06	(0.03–0.08)	0.00	0.17	(0.02–0.32)	0.03
Age started school (years)	-0.02	(-0.04–0.01)	0.01	-0.12	(-0.22–0.02)	0.02
Repeated grades (pp)	-0.03	(-0.13–0.02)	0.17	-0.03	(-0.08–0.03)	0.35
Dropout (pp)	-0.05	(-0.09–0.00)	0.03	-0.03	(-0.07–0.01)	0.09
Years of schooling (years)	0.11	(0.04–0.17)	0.00	0.59	(0.18–1.00)	0.00

All values are marginal effects; 95% of CIs are in parentheses.

^*1*^Height-for-age according to WHO Standard Growth Reference for School-aged Children and Adolescents [37]. Control variables included but not reported are sex, age, parental age, mother working, household size, household’s wealth index, household’s infrastructure (electricity, drinking water, and sanitation) and region (rural/urban areas and fixed effect for main island). We have reported the results using a linear probability model for dichotomous outcomes (repeated education and dropout school). The coefficient x 100 was the marginal effect in percentage points. HAZ: height-for-age z score; IV: instrumental variable; OLS: ordinary least squares.

^2^The details regarding the sample size can be found in S1 Table.

Concerning educational achievement, a one-SD in HAZ in childhood is also significantly associated with longer schooling years (0.11 years) and a later age of first enrolment in school (0.02 years). Moreover, based on linear probit model, being relatively shorter by 1-SD might be related to a higher probability of dropout secondary school (5%), while the relationship between the HAZ and grade repetition was not significant.

The IV result for relative height (Table 3: *4–6*) suggests that being relatively shorter is significantly associated with lower educational achievements. Specifically, a one SD decrease in height is associated with a 3% increase in the probability of dropping out of school, a reduction of approximately 0.6 years in schooling, and a 0.12-year delay in the age of the first enrolment in school. For cognitive skills, a one-SD increase in HAZ is related to an increase of 0.23 and 0.13 z-score points in cognitive and numerical scores in childhood, and the influence persists and declines until adolescence. In contrast, in line with the OLS results, relative height influences numerical abilities more than cognitive ones during adulthood.

We measure two test statistics assessing the strength of the instruments: the Kleibergen-Paap Lagrange Multiplier (LM) and Kleibergen-Paap *F-tests* of weak devices. The Kleibergen-Paap *LM*-tests the null hypothesis that the excluded instruments are correlated with the endogenous variable, and The Kleibergen-Paap *F-test* examines a different null hypothesis relating to weak devices, where weak means having bias relative to the bias in the OLS estimates [20]. The values of the *LM-test* show that we reject the null hypothesis that the excluded instruments are not correlated with the endogenous variable at the *P<0*.*001* level. Similarly, based on the tabulations found in Stock and Yogo (2005) [54], the critical value for the Kleibergen-Paap *F-test* statistic at the 5% significance level is 19.93 for rejecting the null hypothesis of weak instruments, when weak is defined as having a bias in the IV results that is larger than 10% of the bias in the OLS results. Accordingly, we conclude that our instruments have strong explanatory power (S2 Table). In addition, S2 Table reports the *P-values* for the Hansen *J*-statistic for overidentification, where the null hypothesis is that the overidentification constraint is valid, meaning that the model is well-defined. The instrument is not included in the second-stage equation. Failure to reject the null hypothesis for Hansen’s test suggests that all instruments are valid. The instrument set includes birth season and mother height, which gives us some confidence in the power of this specific test. In all cases, we failed to reject null at *P<0*.*05*.

### Stunting and educational outcomes

The results for the relationship between stunting and educational achievement are shown in Table 4. As an indicator of chronic malnutrition, the relationship between stunting and educational attainment increases to nearly three folds the effect of relative height. However, stunting is associated with fewer educational outcomes than relative height.

**Table 4:** Estimation results for stunting and educational outcomes.

	Stunting^1^ when measured prior to age five^2^					
Educational outcomes	OLS	ci	*P*	IV	ci	*P*
	(1)	(2)	(3)	(4)	(5)	(6)
Childhood’s Raven (*Z*-scores)	-0.13	(-0.23–0.04)	0.01	-0.82	(-1.42–0.21)	0.01
Childhood’s Numerical (*Z*-scores)	-0.02	(-0.10–0.06)	0.64	-0.49	(-1.03–0.05)	0.08
Adolescence’s Raven (*Z*-scores)	-0.06	(-0.12–0.01)	0.08	-0.38	(-0.72–0.03)	0.03
Adolescent’s Numerical (*Z*-scores)	-0.02	(-0.09–0.05)	0.63	-0.44	(-0.82–0.06)	0.02
Adult’s Raven (*Z*-scores)	-0.06	(-0.14–0.03)	0.18	-0.07	(-0.63–0.49)	0.80
Adult’s Numerical (*Z*-scores)	-0.17	(-0.26–0.09)	0.00	-0.63	(-1.17–0.08)	0.03
Age started school (years)	0.06	(0.00–0.12)	0.04	0.42	(0.08–0.77)	0.02
Repeated grades (pp)	0.14	(0.00–0.27)	0.05	0.09	(-0.10–0.28)	0.36
Dropout (pp)	0.13	(0.00–0.26)	0.04	0.11	(-0.02–0.23)	0.10
Years of schooling (years)	-0.3	(-0.53–0.08)	0.01	-2.06	(-3.47–0.65)	0.00

All values are marginal effects; 95% of CIs are in parentheses.

^*1*^HAZ below -2 Standard Deviation according to WHO Standard Growth Reference for School-aged Children and Adolescents [37]. Control variables included but not reported are sex, age, parental age, mother working, household size, household’s wealth index, household’s infrastructure (electricity, drinking water, and sanitation) and region (rural/urban areas and fixed effect for main island). We have reported the results using a linear probability model for dichotomous outcomes (repeated education and dropout school). The coefficient x 100 was the marginal effect in percentage points. HAZ: height-for-age z score; IV: instrumental variable; OLS: ordinary least squares.

^2^The details regarding the sample size can be found in S1 Table.

The OLS estimates (Table 4: *1–3*) show that stunting would likely decrease children’s non-verbal abilities (Raven’s test) but not significantly related to numerical scores, except when they grow into adulthood. Stunted children would probably have lower cognitive scores in childhood (0.13-SD) and maturity (0.06-SD). In this phase, being stunted would not likely reduce mathematical scores. However, the influence of stunting on non-verbal cognitive abilities diminishes in adulthood and has a more significant influence on mathematical proficiency. Stunting is also linked to shorter years of schooling (0.3 years) and 0.06 years of later enrolment in school. However, linear probit model shows that stunting has no significant relationship with the probability of repeated grades and dropping out of school.

The IV estimation results suggest that stunting tends to reduce children’s educational achievement and opportunities for higher education (Table 4: *4–6*). Childhood stunting is linked to an around 0.42-year delay in entering school (or five months) and a 2-year reduction in schooling. Regarding cognitive abilities, stunting influences non-verbal skills (Raven test scores) in childhood, but the influence of stunting is strengthened for numerical skills in all three life stages, with the highest impact in adulthood.

We performed test statistics used to assess the strength of two stunting instruments, namely birth season and mother’s height, indicate that both the LM test for endogeneity and the F test for weak devices yield values that lead us to reject the null hypothesis. This means that the excluded instruments are not correlated with the endogenous variable, and the instruments have a strong explanatory power (S3 Table).

## Discussion

Using a longitudinal survey from 1993 to 2014, we examine the correlation between the relative height and stunting status of children under five years old in 1993 or 1997, and their cognitive and educational outcomes at ages 17 to 26 in 2014. This cohort consists of individuals initially included in the study as children and successfully traced into adulthood in at least one wave. During this 21-year period, which encompasses three life phases—school age, adolescence, and adulthood—most participants had either completed high school or were engaged in employment. We apply multivariate regression and instrumental variables models to investigate the potential impact of childhood stunting.

These findings are consistent with previous studies that showed that short stature of children due to childhood stunting at an early age was associated with poor cognitive development later in life, leading to reduced educational outcomes. Specifically, stunting is negatively linked to cognitive abilities [17, 18, 20], lower educational outcomes [15, 20], and delays in enrolling in primary school [20]. The results are significant after adjusting for the confounding effects of age, sex, parental and household characteristics, and regional aspects.

Relative height (HAZ) and stunting significantly influence fluid intelligence during school age. It can be concluded from the study that relative height and stunting have a greater influence on fluid intelligence from school age to adulthood yet, as the individual grows, some abilities, such as numerical ones, could be improved along the way. However, these altered abilities may not compensate for the lagging of educational attainment.

This indicates that stunting and relative height work as an intermediary for cognitive deficits. This is because poor health during childhood potentially contributes to difficulty following formal education; thus, children might have difficulties attending lessons, increasing absenteeism and lacking the energy to learn in the classroom [55].

The results also suggest that the adverse consequences of chronic undernutrition in early life on children’s intellectual development may be exacerbated by environmental factors in the family and/or community, such as the care and affection received from parents. Stunted children may be treated differently from non-stunted children because of their smaller stature and often appearing younger than their age (*Rosenthal effect*) [6, 55], which can affect their abilities and interest in exploring their environment.

Furthermore, this study suggests that chronic malnutrition indirectly correlates with schooling outcomes through decreased cognitive abilities. The effects were significant and relatively stronger in the relationship between stunting and education. Stunted children in this cohort had a marked delay in the first enrolment and a shorter length of schooling, and the magnitude of the relationship between undernutrition and educational achievement in Indonesia is relatively higher compared to other studies. The study found a 0.06-year delay in elementary school enrolment, compared to Victora’s (2008) finding (0.9 years) [15]. As for the length of schooling, by applying a similar method, Hoddinott (2013) reported up to 4.6 years of reduced schooling, while this study found two years shorter [20].

The declining relationship between stunting and cognitive education outcomes with increasing age is likely a result of inadequate policies aimed at reducing stunting and poor education sector performance that fails to provide optimal cognitive development opportunities for non-stunted children [56, 57].

Bogin (2021) [58] argues that social-economic-political-emotional (SEPE) factors influence community views towards adults based on their height which is more visible compared to the more intangible assessment of cognitive ability, which explains the variation of the implication of stunting in the different life course. Another argument stems from the possibility that stunted children may experience growth delays after catching up on their height growth deficit as an opportunity for extended growth due to delayed maturity after puberty [59, 60]. Third, there is debate over the reference data used in measuring stunting. Scheffler and Hermanussen (2021) [61] conducted a historical study on the Indonesian population that showed that the height of Indonesians has never been equal to the European population, which is used as the “normal value” for measuring stunting, making stunting as normal cognition on human height. Therefore, shorter height does not imply a difference in physical fitness among children with stunting [62]. Some studies provide alternative measurements of malnutrition that might comply with the standards in the Indonesian population, such as height-for-difference (HAD) and thus considered a more representative measure [63–66].

Therefore, this study highlights the importance of early intervention, particularly for children nutritionally disadvantaged at age five, mainly due to its detrimental effect on child development. Heckman (2007) suggests that interventions for disadvantaged young children are more effective than those later in life, and remediation at a later age might be costly [67]. However, considering stunting is associated with various socio-cultural and economic disadvantages thus, variations in environments and parenting practices may provide schooling and other learning experiences which may mitigate the effects of early undernutrition on cognition [17, 54]. Ensuring children with early stunting receive schooling comparable in quantity and quality to non-stunted children could help improve their educational outcomes.

This implies policy responses that require the involvement of various parties at different levels and the identification of actors needed to encourage changes at the community and household levels, particularly during children’s early years. Indonesia has committed to investing significant resources, equivalent to USD51.9 trillion, in cross-sectoral strategies to address stunting [68]. Food policy, equitable distribution of health provision at the village level, conditional social assistance, clean water and sanitation infrastructure have been identified as the most effective strategies for improving stunting rates and overall health quality in Indonesia especially for the poor [24, 26, 69, 70]. The study also indicates the importance of household wealth and parental education in children’s nutritional status and educational outcomes, implying that policies to improve households’ livelihood would positively affect children’s nutrition and education.

Our findings may be limited by the substantial level of attrition and exclusion of the variables that may affect relative height and education. Even though applying 2SLS with instrument variables is considered the optimal effort to encounter the potential problems, it can be challenging and hard to verify. Despite these challenges, the instrumental variable tests conducted in this study are suitable. Meanwhile, this study’s strength is filling the literature gap on the implications of stunting in Indonesia. Studies in similar areas have been conducted in various countries, but none have been done specifically for the case of Indonesia. Yet, Indonesia is a country with one of the largest populations and an economy that is considered globally significant.

## Conclusion

Our study shows a strong relationship between stunting and lower cognitive abilities that is likely to persist and lead to lower educational outcomes over the long term. However, the relationship appears to weaken as individuals enter adulthood, potentially indicating the influence of environmental factors. This finding suggests that recent development has not provided an adequate environment for children to reach their academic potential, potentially leading to a decline in future labour quality. To address this issue, it is necessary to prioritise addressing stunting and its underlying determinants, including social and economic factors. This will require collaborative efforts from various parties to address the causes of stunting and reduce its prevalence.

## Supporting information

S1 Table Sample sizes for the regression results of the relationship between HAZ and stunting on educational and cognitive achievements. (DOCX)

S2 Table Instrumental variables test statistics by domain for HAZ. Note: ^1^. *** is significant at 95%. ^2.^ Stock-Yogo critical values alpha = 5%; Bias 10%, two instruments: 19.93; Bias 15%, two instruments: 11.59.
(DOCX)

S3 Table Instrumental variables test statistics by domain for STUNTING. Note: ^1^. *** is significant at 95%. ^2.^ Stock-Yogo critical values alpha = 5%; Bias 10%, two instruments: 19.93; Bias 15%, two instruments: 11.59.
(DOCX)

## List of abbreviations

IFLS1-5: Indonesia Family Life Survey waves 1 to
HAZ: Height-for-age z-score
WHO: World Health Organization
PCA: Principal component analysis
IVs: Instrumental variables
OLS: Ordinary least squares
SD: Standard deviation
2SLS: two stages least square
