# Supplementary material for: Stunting and its association with education and cognitive outcomes in adulthood: A longitudinal study in Indonesia
Source: PLoS One. 2024 May 6;19(5):e0295380. doi: 10.1371/journal.pone.0295380 (PMC11073707; doi:10.1371/journal.pone.0295380)
Supplement: S1 Table — (DOCX) [file pone.0295380.s001.docx]

**S1 Table. Sample Sizes for the regression results of the relationship between HAZ and stunting on educational and cognitive achievements**

|  | HAZ | | Stunting | |
| --- | --- | --- | --- | --- |
| VARIABLES | OLS | IV | OLS | IV |
| Childhood's Raven (*Z*-scores) (wave 3/2000) | 1,755 | 1,585 | 1,587 | 1,587 |
| Childhood's Numerical (*Z*-scores) (wave 3/2000) | 1,755 | 1,477 | 1,587 | 1,587 |
| Adolescence's Raven (*Z*-scores) (wave 4/2007) | 2,778 | 2,492 | 2,498 | 2,498 |
| Adolescent's Numerical (*Z*-scores) (wave 4/2007) | 2,778 | 2,436 | 2,498 | 2,498 |
| Adult's Raven (*Z*-scores) (wave 5/2014) | 1,986 | 2,076 | 1,802 | 1,802 |
| Adult's Numerical (*Z*-scores) (wave 5/2014) | 1,986 | 2,298 | 1,802 | 1,802 |
| Age started school (years) (wave 5/2014) | 2,410 | 2,165 | 2,165 | 2,165 |
| Repeated grades (pp) (wave 5/2014) | 2,111 | 1,915 | 1,915 | 1,915 |
| Dropout (pp) (wave 5/2014) | 3,066 | 2,752 | 2,752 | 2,752 |
| Years of schooling (years) (wave 5/2014) | 2,444 | 2,195 | 2,195 | 2,195 |
