# Supplementary material for: Stunting and its association with education and cognitive outcomes in adulthood: A longitudinal study in Indonesia
Source: PLoS One. 2024 May 6;19(5):e0295380. doi: 10.1371/journal.pone.0295380 (PMC11073707; doi:10.1371/journal.pone.0295380)
Supplement: S2 Table — Note: 1. *** is significant at 95%. 2. Stock-Yogo critical values alpha = 5%; Bias 10%, two instruments: 19.93; Bias 15%, two instruments: 11.59. (DOCX) [file pone.0295380.s002.docx]

**S2 Table. Instrumental variables test statistics by domain for HAZ.**

| **Educational Outcomes** | **Kleibergen-Paap**  ***LM-stat*^1^** | **Kleibergen-Paap**  ***F-stat*^2^** | **Hansen J stat**  ***P*-value1** |
| --- | --- | --- | --- |
| Childhood’s Raven (*Z*-scores) | 58.38*** | 32.19 | 0.84 |
| Childhood’s Numerical (*Z*-scores) | 54.77*** | 30.85 | 0.30 |
| Adolescence’s Raven (*Z*-scores) | 88.43*** | 50.44 | 0.05** |
| Adolescent’s Numerical (*Z*-scores) | 83.88*** | 47.52 | 4.39 |
| Adult’s Raven (*Z*-scores) | 68.54*** | 38.14 | 0.00 |
| Adult’s Numerical (*Z*-scores) | 75.59*** | 43.95 | 0.01 |
| Age started school (years) | 70.14*** | 39.10 | 0.90 |
| Repeated grades (pp) | 58.24 | 33.07 | 4.34 |
| Dropout (pp) | 98.80 | 55.99 | 0.05 |
| Years of schooling (years) | 70.93 | 39.30 | 0.93 |

Note: ^1^. *** is significant at 95%.

^2.^ Stock-Yogo critical values alpha=5%; Bias 10%, two instruments: 19.93; Bias 15%, two instruments: 11.59.
