# Supplementary material for: Stunting and its association with education and cognitive outcomes in adulthood: A longitudinal study in Indonesia
Source: PLoS One. 2024 May 6;19(5):e0295380. doi: 10.1371/journal.pone.0295380 (PMC11073707; doi:10.1371/journal.pone.0295380)
Supplement: S3 Table — Note: 1. *** is significant at 95%. 2. Stock-Yogo critical values alpha = 5%; Bias 10%, two instruments: 19.93; Bias 15%, two instruments: 11.59. (DOCX) [file pone.0295380.s003.docx]

**S3 Table. Instrumental variables test statistics by domain for STUNTING.**

| **Educational Outcomes** | **Kleibergen-Paap**  ***LM-stat*^1^** | **Kleibergen-Paap**  ***F-stat*^2^** | **Hansen J stat**  ***P*-value^1^** |
| --- | --- | --- | --- |
| Childhood's Raven (*Z*-scores) | 41.67*** | 21.50 | 0.02 |
| Childhood's Numerical (*Z*-scores) | 41.71*** | 21.49 | 0.43 |
| Adolescence's Raven (*Z*-scores) | 87.89*** | 46.15 | 3.45* |
| Adolescent's Numerical (*Z*-scores) | 82.26*** | 43.30 | 0.65 |
| Adult's Raven (*Z*-scores) | 61.82*** | 32.15 | 0.00 |
| Adult's Numerical (*Z*-scores) | 71.96*** | 37.63 | 0.02 |
| Age started school (years) | 67.92*** | 35.43 | 0.88 |
| Repeated grades (pp) | 58.73*** | 30.62 | 0.04 |
| Dropout (pp) | 93.26*** | 48.66 | 0.71 |
| Years of schooling (years) | 66.24*** | 34.51 | 0.35 |

Note: ^1^. *** is significant at 95%.

^2.^ Stock-Yogo critical values alpha=5%; Bias 10%, two instruments: 19.93; Bias 15%, two instruments: 11.59.
